# A Novel High-Density Phase and Amorphization of Nitrogen-Rich 1H-Tetrazole (CH_2_N_4_) under High Pressure

**DOI:** 10.1038/srep39249

**Published:** 2017-02-20

**Authors:** Wenbo Li, Xiaoli Huang, Kuo Bao, Zhonglong Zhao, Yanping Huang, Lu Wang, Gang Wu, Bo Zhou, Defang Duan, Fangfei Li, Qiang Zhou, Bingbing Liu, Tian Cui

**Affiliations:** 1 State Key Laboratory of Superhard Materials, College of physics, Jilin University, Changchun, 130012, P. R. China

## Abstract

The high-pressure behaviors of nitrogen-rich 1H-tetrazole (CH_2_N_4_) have been investigated by *in situ* synchrotron X-ray diffraction (XRD) and Raman scattering up to 75 GPa. A first crystalline-to-crystalline phase transition is observed and identified above ~3 GPa with a large volume collapse (∼18% at 4.4 GPa) from phase I to phase II. The new phase II forms a dimer-like structure, belonging to *P*1 space group. Then, a crystalline-to-amorphous phase transition takes place over a large pressure range of 13.8 to 50 GPa, which is accompanied by an interphase region approaching paracrystalline state. When decompression from 75 GPa to ambient conditions, the final product keeps an irreversible amorphous state. Our ultraviolet (UV) absorption spectrum suggests the final product exhibits an increase in molecular conjugation.

Interest in polymeric nitrogen and nitrogen-rich compounds has evoked great attention to finding materials with high energy and high density^1^,^2^,^3^,^4^,^5^,^6^,^7^,^8^. At present, the polymeric nitrogen network has been successfully synthesized under high pressure and high temperature by Eremets *et al*.^9^ However, its practical application has come into question, because it would transform back to a molecular phase at 42 GPa^10^,^11^,^12^,^13^,^14^,^15^,^16^,^17^. 1H-tetrazole (CH_2_N_4_) is a promising energetic organic material, with high nitrogen content, high formation heat, and good thermal stability^18^. It is expected that this typical nitrogen-rich monomer under high pressure would polymerize more readily than previous studies with nitrogen or azides^9^,^16^. Thus, characterizing and understanding the behaviors of 1H-tetrazole under high pressures are of great importance for seeking the effect of pressure on polymerization of nitrogen-rich molecular solids. In addition, the pressure-induced substantial changes in microscopic level can also directly affect the macroscopic properties, such as the important physical parameter—density. The elevated density upon compression plays an important role in the performance of an energetic material. In a certain pressure range, the higher density an energetic material has, the larger detonation velocity and pressure it may exhibit^19^,^20^. In this framework, if there should exist a large increase of density in 1H-tetrazole, this compound would greatly improve its performance and application, or even if there should be any pressure driving this high-nitrogen compound polymeric, it could be a possible future energetic material.

Figure 1a shows the basic geometry information of 1H-tetrazole. At ambient conditions, in Fig. 1b and c, the solid 1H-tetrazole is triclinic symmetry with a single molecule in a unit cell^21^. The individual molecules connect each other with N–H•••N bridges and chain each other, which further connect by C–H•••N bridges to form a two-dimensional network or sheet in Fig. 1d. The distance between neighboring sheets equals to that in graphite, 3.35 Å. Such a unique structure makes 1H-tetrazole a good candidate for insensitive high explosive (IHE). Up to now, research on 1H-tetrazole under extreme conditions is still lacking. Consequently, the direct observation from high-pressure experimental measurement is extremely necessary.

In this work, *in situ* high-pressure synchrotron X-ray diffraction (XRD) and Raman scattering spectra have been performed to investigate the behaviors of 1-H tetrazole. A series of novel phenomena have been found, involving a crystalline-to-crystalline transition and a crystalline-to-amorphous transition. The crystalline-to-crystalline transition takes place with a large volume collapse (∼18% at 4.4 GPa), and forms a high-density phase. The crystalline-to-amorphous transition is accompanied by an interphase region approaching paracrystalline state over a large pressure range. However, when pressure exceeds a threshold, an irreversible transformation occurs. The final product released from that pressure keeps amorphous state, and its ultraviolet (UV) absorption spectrum suggests the product exhibits an increase in molecular conjugation.

## Results and Discussion

Figure 2a shows the typical XRD patterns of 1H-tetrazole with increasing pressure below ~11 GPa. The primary diffraction peaks at ambient pressure are indexed based on the reported structure^21^ we note as phase I. All peaks shift to higher 2*θ* angles with pressure increase, due to the contraction of cell volume and corresponding *d*-spacings. At 3.1 GPa, new peaks marked with asterisks emerge, with the relative intensities growing from 3.1 to 6.5 GPa, whereas the original peaks (001), (010), (01-1), (100), (10-1) in phase I weaken and completely vanish at 7.8 GPa. These obvious phenomena reveal that a crystalline-to-crystalline transition occurs at 3.1 GPa then completes at 7.8 GPa. The coexistence region from 3.1 to 7.8 GPa is largely caused by slow transition kinetics^22^,^23^,^24^. At 7.8 GPa, the sample is fully converted into a new phase, and no structural changes are observed in the XRD profiles up to ~11 GPa. At 13.8 GPa in Fig. 2b, small halo emerges in the XRD pattern, superimposing on the sharp diffraction peaks in the range of 8.3°–9.4°, while the sharp diffraction peaks begin to weaken with elevated pressure. This phenomenon seems to be a coexistence state accompanied by an interphase region approaching paracrystalline state^25^,^26^. At ~30 GPa, two haloes are left with sharp peaks vanishing. Up to ~50 GPa, the crystalline state has completely transformed into amorphous state, evidencing a broad peak. When decompression from 75 GPa to ambient conditions, product keeps amorphous state.

**Figure 1:**
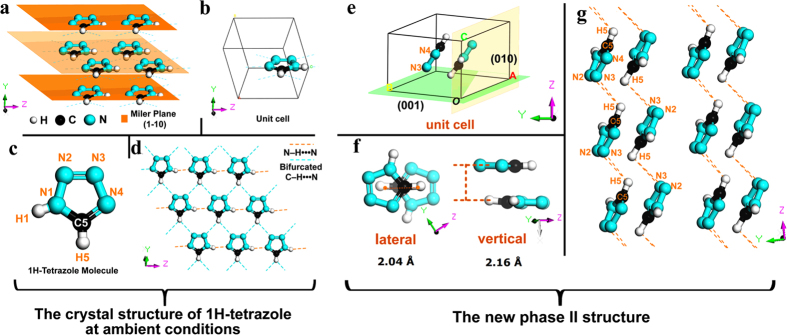
The crystal structure of 1H-tetrazole. (**a**) Triclinic symmetry in sheets. The distance between neighboring sheets equals to that in graphite, 3.35 Å. (**b**) A single molecule in a unit cell. (**c**) 1H-tetrazole molecule with atom numbering. (**d**) Typical 2D network sheet with hydrogen bonds in (1-10) plane. Dashed lines represent intermolecular hydrogen bonds: N–H···N and bifurcated C–H···N. (**e**) Unit cell of phase II at 7.8 GPa. Green part represents (001) plane and yellow part (010) plane. (**f**) The lateral offset and vertical distance in the dimer-like structure at 7.8 GPa. (**g**) The hydrogen-bond architecture in phase II. The distances of C5H5•••N2 and C5H5•••N3 are about 3.4 Å. The dash lines represent the hydrogen-bond interactions.

**Figure 2:**
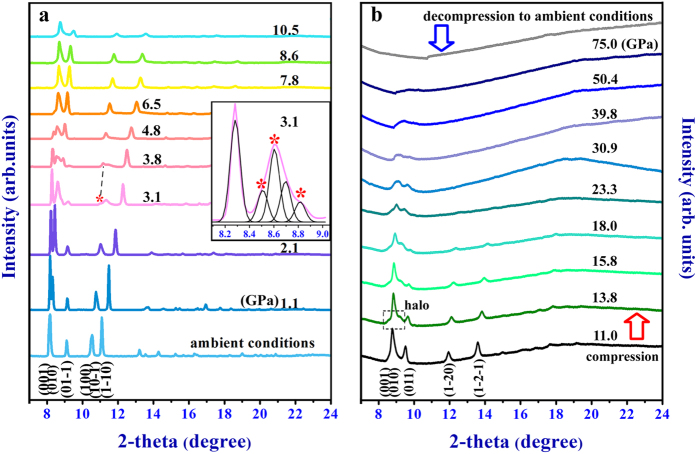
X-ray diffraction measurements (incident wavelength λ = 0.6199 Å) of 1H-tetrazole. (**a**) Representative X-ray diffraction patterns below ~11 GPa. The asterisks marked on the XRD patterns represent peaks from new phase. (**b**) X-ray diffraction patterns from 11 to 75 GPa and after pressure release. At 13.8 GPa, dotted box is halo part. Up to 50 GPa, only a broad peak is left.

**Figure 3:**
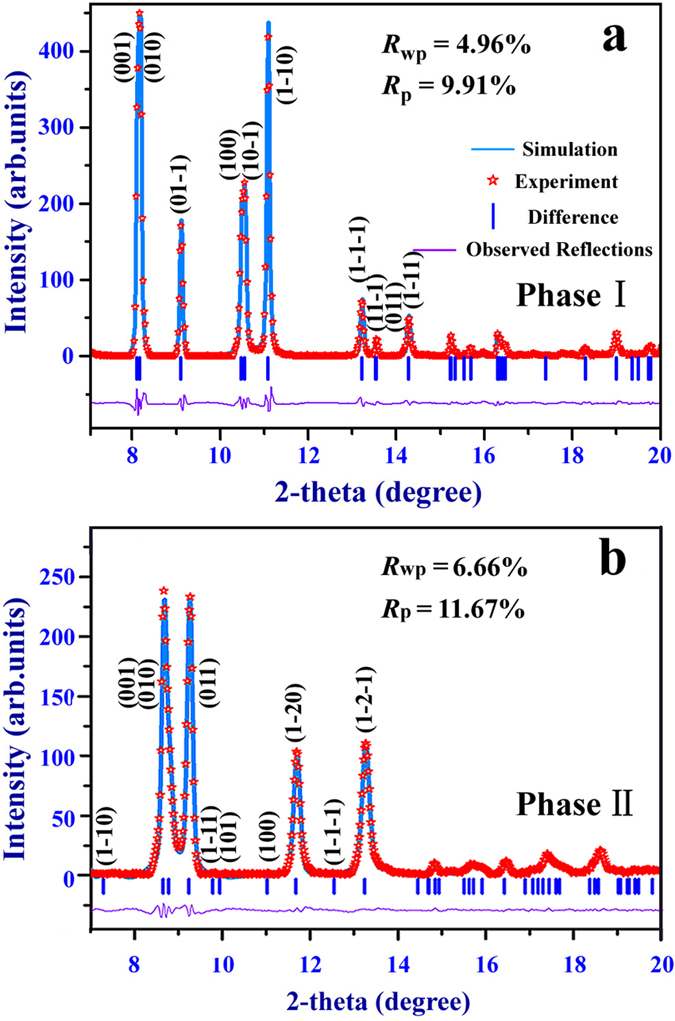
Rietveld full-profile refinement of the XRD patterns. (**a**) Phase I at ambient conditions. (**b**) New phase II at 7.8 GPa. The two fits are good for the diffraction patterns shown with *R*_wp_ = 4.96%, *R*_p_ = 9.91% for phase I and *R*_wp_ = 6.66%, *R*_p_ = 11.67% for phase II.

It is of fundamental interest to probe the pressure-induced new solid phases. At ambient conditions, the Rietveld refinement of XRD pattern shows a good agreement with phase I structure^21^ in Fig. 3a. Primary diffraction peaks are well indexed. The refined lattice parameters are *a* = 3.7370 (2) Å, *b* = 4.7818 (3) Å, *c* = 4.9443 (4) Å, *α* = 106.977 (9)°, *β* = 107.17 (1)°, *γ* = 101.715 (7)° with unit cell volume *V* = 76.58 (2) Å^3^. The X-ray results provide clear evidence for pressure-induced phase transition in 1H-tetrazole below 10 GPa. However, the structure of the new phase is still unknown. By using the Reflex Module combined in Materials Studio program^27^, the XRD pattern at 7.8 GPa is best indexed with triclinic symmetry. After comparing the simulated XRD patterns from the possible triclinic structures with our experimental XRD, the Pawley refinement results suggest the symmetry of new phase also belongs to *P*1 space group. Then two non-equivalent molecules CH_2_N_4_ are added into the *P*1 structure. By means of Rietveld refinement, the most likely structure of high-pressure phase is obtained in Fig. 1e. Here we note it as phase II. The result of Rietveld refinement is given in Fig. 3b. The refined lattice parameters are *a* = 5.229 (3) Å, *b* = 6.307 (1) Å, *c* = 4.881 (3) Å, *α* = 84.71 (2)°, *β* = 72.20 (3)°, *γ* = 133.41 (1)° with unit cell volume *V* = 100.49 (1) Å^3^. The values of *R*_p_ and *R*_wp_ suggest the acceptability of the proposed structure and the atomic coordinates of phase II structure at 7.8 GPa are listed in Table 1.

Comparing with phase I, the new phase II in Fig. 1e is a high-density stacking at 7.8 GPa, which presents an increase in the number of molecules in a unit cell. A pair of antiparallel rings, in a unit cell, is stacked forming a dimer-like configuration with an offset or slipped packing. The lateral offset of centers and the vertical distance of the two rings are respectively ~2.04 Å and ~2.16 Å at 7.8 GPa in Fig. 1f, then ~1.82 Å and 2.1 Å at 1 GPa. In addition, after the first phase transition (phase I→II), the original hydrogen bonds with a two-dimensional network are thoroughly broken up and rearranged. In new phase II (see Fig. 1g), the distances of C5H5•••N2 and C5H5•••N3 between the neighboring dimer-likes are about 3.4 Å (in the range of hydrogen bond)^28^, thus the C5H5•••N2 and C5H5•••N3 in phase II might form bifurcated hydrogen-bond interactions. The feature of hydrogen-bond configuration in phase II exhibits as a wave shape along *c*-axis, different from the hydrogen-bond connection type in phase I. From ~13.8 to 50 GPa is a crystalline-to-amorphous transition. According to our phase II structure with dimer-like configuration, the repulsive force occurring between π-π stacking may make the molecules distortion, and part of lattice displays a degree of local disorder. Thus, the fluctuations of the lattice spacing generate the phase difference (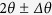) of the same (*hkl*), then haloes appear^29^,^30^.

**Figure 4:**
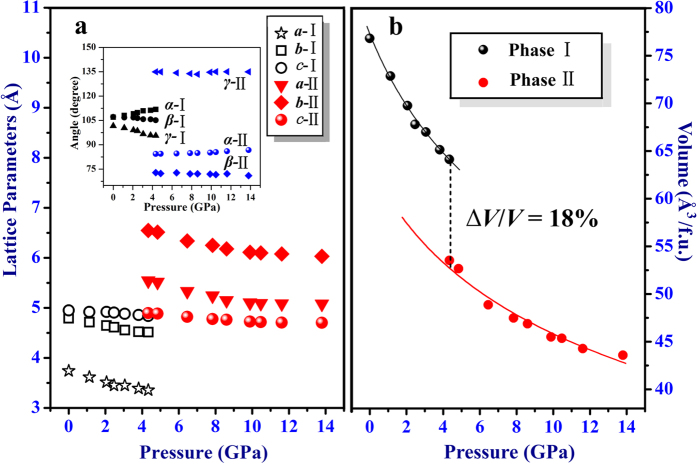
The lattice parameters and volumes. (**a**) Lattice parameters at different pressure in phase I and II. (**b**) Pressure-volume data of phase I and II at 300 K. Black and red symbols represent experiment data, and solid black lines are third-order Birch-Murnaghan equation of state fitting data.

Figure 4a shows the respective lattice parameters of phase I and II as a function of pressures. It is observed that the lattice parameters (*a, b, c*) in two phases decrease monotonically with increasing pressure except angles. The volume per unit as a function of pressure is fitted by Birch-Murnaghan (BM) equation of state (EOS)^31^ in Fig. 4b,


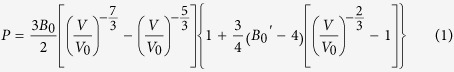


where *V*_0_ is the volume per formula unit, *V* is the volume per formula unit at pressure *P* given in GPa, *B*_0_ is the isothermal bulk modulus, and *B*_0_′ is the first pressure derivative of the bulk modulus. In our experimental data, *B*_0_′ is set as 4. From the fitting results, it is found that the average volume occupied by each molecule is largely reduced by ~18% at 4.4 GPa during the crystalline-to-crystalline phase transition. Consequently, the new phase II is a high-density stacking, which might be a novel structure type for nitrides. The large increase of density at a lower pressure might greatly enhance the performance and application of 1H-tetrazole in energetic materials^20^.

At ambient conditions, the predominant cohesive factors in 1H-tetrazole are hydrogen bonds (C–H···N, N–H···N) and van der Waals interactions that lead to a large proportion of empty space left in phase I. At the onset of compression, the interlayer distance reduces faster than the distance between molecules in the layers. Upon further compression to ~3.1 GPa, the planar hydrogen-bond network can no longer support the increased energy of interlayer interactions. This can give rise to the corrugation of the molecular sheet. Subsequently, phase transition and rearrangement of hydrogen-bond networks occur, in order to reduce the Gibbs free energy^32^,^33^. The pressure-induced crystalline-to-crystalline transition mechanism for 1H-tetrazole have some similarities with the layered adduct formed by cyanuric acid and Melamine (CA·M)^34^. After all, 1H-tetrazole is of aromatic compound. The classical example of aromatic compound is benzene. With pressure increase, benzene can transform into a configuration resembling dimer-like motif before amorphization as 1H-tetrazole^35^,^36^. Likewise, the *P*2_1_/*c* phase of pyrrole (C_4_H_5_N) also forms dimer-like motif after crystalline-to-crystalline transition as 1H-tetrazole^37^. The dimer-like motif seems to be a feature of certain aromatic compounds before pressure-induced amorphization. And the pressure-induced amorphous phenomena in aromatic compounds are actually common as well, such as pyridine, furan, pyrimidine, s-triazine, etc.^38^,^39^,^40^.

**Figure 5:**
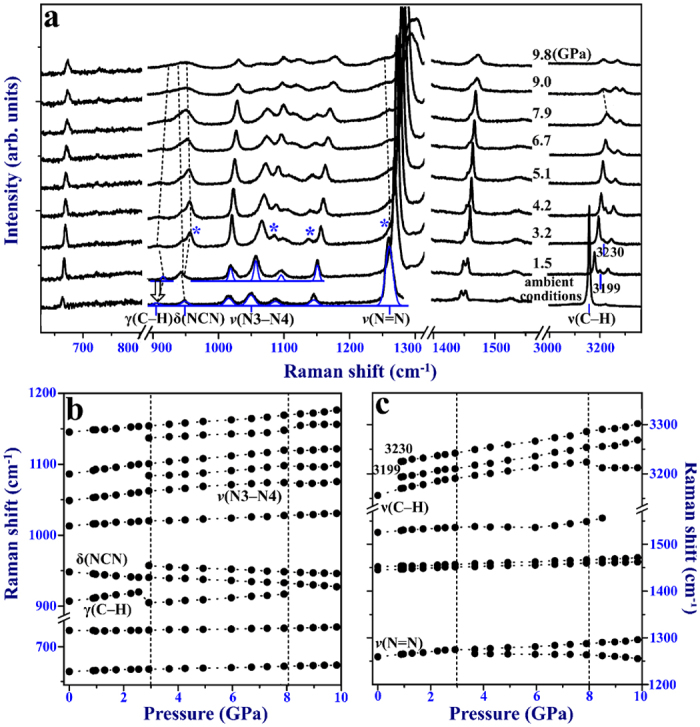
Raman scattering in internal modes region. (**a**) Typical Raman spectra up to ~10 GPa. New emerging modes are marked with asterisks. (**b**) and (**c**) Raman shifts with increasing pressure. Two dashed lines suggest the pressure range of phase transition.

To comprehensively investigate the behaviors of 1H-tetrazole under high pressures, Raman spectrum is also a vital tool. Figure 5a shows a complete picture of internal modes with increasing pressure below ~10 GPa. Table 2 lists our observed internal modes at ambient conditions, which agrees well with previous studies^41^,^42^. At 3.2 GPa, some new peaks (956, 1083, 1138, and 1247 cm^−1^) emerge, splitting from the major peaks 946, 1065, 1152, and 1288 cm^−1^, which suggests the onset of phase transition. All these splittings in internal modes are believed to arise from interactions between crystallographically inequivalent molecules, as Davydov splitting^43^. In many cases, Davydov splitting is related to a possible increase in the number of molecules in a unit cell as well as associated symmetry lowering^44^,^45^,^46^. Thus, our Raman spectra indirectly identify the phase transition forming a pair of molecules in a unit cell of phase II. According to group theory, phase II belongs to low symmetry *P*1 with a total of 14 atoms in a unit cell. There should exist 39 Raman active modes.

Figure 5b and c show the nonlinear Raman shifts dependent on pressure in the internal modes region. All modes shift gradually toward higher frequencies with increasing pressure except some special modes displaying red shift. The increase in frequencies of modes could be explained by the decrease of interatomic distances and the increase in the effective force constants with pressure. The obvious discontinuous range from ~3 to 8 GPa suggest the phase transition pressure range. There are some special modes needed to note. First, the in-plane bending mode δ(NCN) at ~947.6 cm^−1^ shows a red shift upon compression, different from other modes with a blue shift. Consequently, we compress and refine the phase I structure of 1H-tetrazole at ambient conditions, 1.1 GPa and 2.1 GPa by using Materials Studio. And the bond length of N=C stretches from 1.315 Å to 1.322 Å, which may be the reason for the red shift of δ(NCN) mode with pressure increase. Second, the out-of-plane bending mode γ(C–H) occurs frequency drop at ~3.2 GPa. This phenomenon seems to be also related to the change of bond length. From ambient conditions to 2.1 GPa, the bond length of C–H shortens from 0.874 Å to 0.760 Å. However, up to 3.1 GPa, the bond length of C–H stretches to 0.934 Å. Thus, it is the increase of interatomic distances that causes a slight frequency drop of the γ(C–H) mode, which suggests the starting of the phase transition process. Additionally, a new emerging mode 1247 cm^−1^ splitting from *ν*(N=N) mode shows a red shift above 3.2 GPa. Above 300 cm^−1^, the prominent mode 3157.1 cm^−1^ at ambient pressure is assigned to *ν*(C–H) stretching mode^41^. This *ν*(C–H) mode participates in a special bifurcated hydrogen-bond (C–H···N), as shown in Fig. 1d. Unlike other hydrogen bonds showing a red shift with increasing pressure, this bifurcated hydrogen bonds C–H···N are found to display a blue shift below ~7.8 GPa. Li’s study^47^ reports that the attractive and repulsive interactions coexist in the proton donor and acceptor of hydrogen-bond system. The proportion of attractive and repulsive interactions determines the red or blue shift of hydrogen bonds. Here the blue shift of bifurcated hydrogen-bond C-H•••N on compression is considered that the repulsive forces cause the C–H shortening, overwhelming the elongation caused by the attractive interactions. However, at about ~7.9 GPa, the mode *ν*(C–H) at 3227 cm^−1^ abruptly displays a red shift, which indicates that the *ν*(C–H) stretching mode undergoes significant modifications after phase I completely transforming into phase II. The *ν*(N–H) mode is reported to be relatively much less intense at ambient conditions. The visible peaks 3199 and 3230 cm^−1^ at 1.5 GPa might be the results of multiple Fermi resonance interactions with overtones and combination tones^41^.

**Figure 6:**
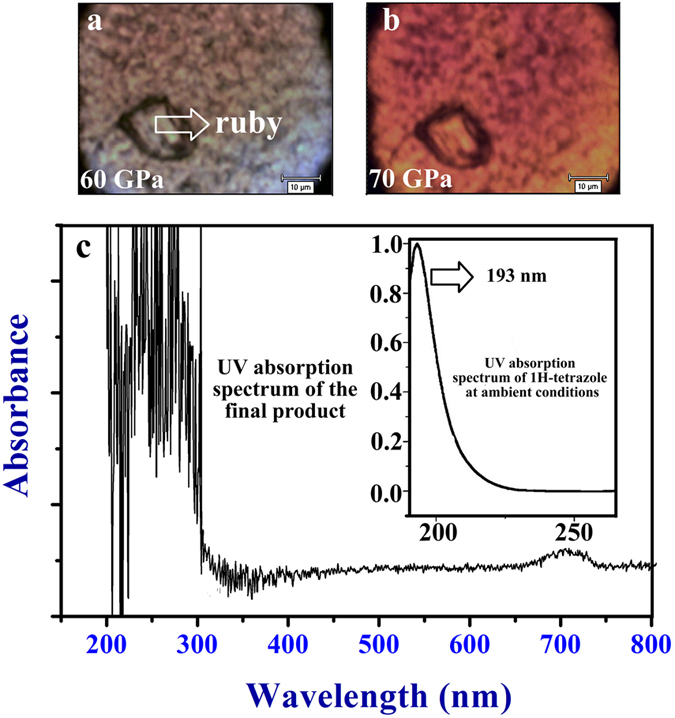
Surface in sample chamber and UV absorption spectrum. (**a**) Sample at 60 GPa. (**b**) Sample at 70 GPa. (**c**) The UV absorption spectrum of our recovered product. Inset is from ref. 50.

When pressure increases to ~60 GPa, we find a color transition on the sample surface in Fig. 6a. For this phenomenon, we perform two sets of compression−decompression cycle on 1H-tetrazole by Raman scattering. The first set of Raman spectrum is reversible (see Figure S1) when decompression from the maximum pressure 45 GPa (white sample at 45 GPa). But the second set is irreversible with high fluorescence background when decompression from ~70 GPa (red sample at 70 GPa). Hence, we conclude that an irreversible transformation in 1H-tetrazole should take place in the pressure range of ~45 to 70 GPa. On studies of benzene, the onset of pressure-induced chemical reaction (polymerization) is caused by π-π overlap of rings^35^,^36^, which provides a foundation for reasoning about 1H-tetrazole. At ~60 GPa, the onset of color change in 1H-tetrazole (white→reddish) indicates a pressure-induced band gap closure. The band gap closure of 1H-tetrazole may result from the close π-π overlap of nitrogen-rich rings, which may simultaneously induce the onset of chemical reaction as benzene. Above 60 GPa, the color of sample amorphous phase intensifies (reddish→red) upon compression (see Fig. 6b), which may suggest a further chemical reaction with an increasing number of conjugated double bonds in system^48^,^49^. Thus, the irreversible product is formed. To further explore the recovered amorphous product from 75 GPa, we have measured UV absorption spectrum in Fig. 6c. Comparing with the reported 1H-tetrazole with absorption peak at 193 nm^50^, the recovered product shows its absorption edge at about 325 nm and a weak absorption peak at 700 nm. By analyzing the characteristic of our absorption spectrum, there should be more n → π* transition in molecular structure of product, which indicates that the amorphous product exhibits an increase in molecular conjugation, possibly polymeric. In fact, studies by Zharov have shown that most molecules containing the groups –C=C–, –C=N–, heterocyclic rings, or aromatic rings can polymerize into a macromolecular under the effect of pressures^51^.

## Conclusion

In conclusion, our experimental results have identified the nitrogen-rich 1H-tetrazole (CH_2_N_4_) undergoes several transitions with elevated pressure. A crystalline-to-crystalline phase transition starts at 3.1 GPa, then ends up at 7.8 GPa with a large volume collapse (∼18% at 4.4 GPa) from phase I to phase II. Phase II is a high-density stacking with *P*1 symmetry. This discovery is caused by structural repacking from layered packing to dimer-like configuration. Subsequently, a crystalline-to-amorphous phase transition takes place over a large pressure range of ~13.8 to 50 GPa, which is accompanied by an interphase region approaching paracrystalline state. This amorphization is considered a gradual lattice and molecular distortion between π-π stacking on compression. Up to 60 GPa, sample shows a color change, which may represent a pressure-induced chemical transformation in amorphous phase. When pressure is completely released from 75 GPa to ambient conditions, the product keeps amorphous phase. Our UV absorption spectrum suggests the amorphous product exhibits an increase in molecular conjugation, possibly polymeric. This study provides new ideas for exploring the synthesis of material with high energy and high density combining nitrogen-rich heterocyclic or aromatic compounds by high-pressure techniques.

## Methods

1H-Tetrazole (98%) is purchased from Sigma-Aldrich Corp. We carry out our high-pressure study with diamond anvil cells (DAC) whose culet diameter of the diamond anvils is 300 *μ*m. The T301 stainless steel gaskets are preindented to a thickness of 40 *μ*m with a center hole of 80 *μ*m. Ruby chips are used for pressure determination by measuring the optical shift of ruby *R*1 fluorescence line^52^. Pressure transmission medium (PTM) is not used during experiment, because 1H-tetrazole is a very soft crystal, which can keep the sample chamber a quasi-hydrostatic condition at high pressures.

The Raman spectra are measured by an ActonSpctraPro500i spectrograph with a liquid-nitrogen-cooled CCD detector (Princeton Instruments, 1340 × 100). A solid-state, diode-pumped, frequency-doubled Nd: vanadate laser (*λ* = 532 nm) is used as the exciting laser. Raman spectra are collected in a back scattering geometry with a 1800 gr/mm holographic grating, and the slit width is selected as 80 *μ*m, corresponding to a resolution of ~2 cm^−1^. The sample image can be collected through an achromatic lens and then focused onto a CCD detector for visual monitoring during experiments.

*In situ* angle-dispersive X-ray diffraction (ADXRD) experiments are carried out at 4W2 High Pressure Station of Beijing Synchrotron Radiation Facility (BSRF). Monochromatic wavelength of 0.6199 Å is used for data collection, and the beam spot size is ~20 × 30 *μ*m^2^. The sample−detector distance and geometric parameters are calibrated using a CeO_2_ standard. The Bragg diffraction rings are recorded with an image plate area detector (Mar345) and converted into plots of intensity versus 2*θ* with Fit2D software^53^. Materials Studio 7.0 software (Accelrys Inc.) is used to perform further analysis of XRD data^54^. The UV absorption spectrum is monitored by a Shimadzu UV–3150 spectrometer.

## Additional Information

**How to cite this article**: Li, W. *et al*. A Novel High-Density Phase and Amorphization of Nitrogen-Rich 1H-Tetrazole (CH_2_N_4_) under High Pressure. *Sci. Rep.* **7**, 39249; doi: 10.1038/srep39249 (2017).

**Publisher's note:** Springer Nature remains neutral with regard to jurisdictional claims in published maps and institutional affiliations.

## Supplementary Material

Supplementary Information

## Figures and Tables

**Table 1:** Atomic coordinates in new phase II at 7.8 GPa (Å).

Label	x	y	z
C5	0.481 (2)	0.604 (2)	0.652 (1)
N1	0.777 (1)	0.871 (1)	0.375 (1)
N2	0.606 (6)	0.872 (3)	0.215 (3)
N3	0.207 (4)	0.604 (1)	0.400 (1)
N4	0.118 (6)	0.430 (1)	0.676 (2)
H1	0.062 (3)	0.043 (2)	0.299 (6)
H5	0.514 (1)	0.546 (6)	0.823 (3)
C5	0.537 (1)	0.387 (1)	0.346 (4)
N1	0.220 (3)	0.116 (2)	0.621 (1)
N2	0.358 (1)	0.091 (3)	0.801 (3)
N3	0.758 (5)	0.349 (1)	0.632 (3)
N4	0.880 (5)	0.540 (1)	0.345 (4)
H1	0.970 (1)	0.985 (2)	0.682 (2)
H5	0.527 (1)	0.487 (1)	0.163 (6)

**Table 2:** Assignments and frequencies of vibration modes for 1H-tetrazole (cm^−1^).

*ν*_IR_^*a*^	*ν*_R_^*a*^	*ν*_R_^*b*^	Approximate description^*a*^
884.4	n.o.	n.o.	*γ*(N–H)
663.1	663.7	663.9	*γ*(ring)
722.4	n.o.	723	*γ*(ring)
907.2	905.4	907	*γ*(C–H)
937.1	n.o.	n.o.	*δ*(NNN as.)
951.5	947.2	947.6	*δ*(NCN)
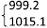	1013.7	1015	*ν*(N1–N2)
1049.6	1048.6	1049	*ν*(N3–N4)
1085.3	1086.1	1086.1	*δ*(C–H)
1145.1	1144.5	1144.8	*δ*(N–H) + *ν*(C=N)
1259.3	1259.9	1259.9	*ν*(N=N)
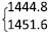	1448.9	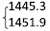	*ν*(C–N)
1524.6	1530.3	1526.3	*ν*(C=N) + *δ*(N–H)
3158.3	3158.5	3157.1	*ν*(C–H)
^*d*^	^*d*^	^*d*^	*ν*(N–H)

^a^Ref. 36.^b^our experimental measuremen. ^c^ν: stretching. δ: in-plane bending. γ: out-of-plane bending; as.: asymmetric; n.o.: not observed. ^*d*^Very broad and complex band.
