# Supplementary material for: A Novel High-Density Phase and Amorphization of Nitrogen-Rich 1H-Tetrazole (CH2N4) under High Pressure
Source: Sci Rep. 2017 Feb 20;7:39249. doi: 10.1038/srep39249 (PMC5316957; doi:10.1038/srep39249)
Supplement: Supplementary Information [file srep39249-s1.doc]

**Supplementary Information**

A Novel High-Density Phase and Amorphization of Nitrogen-Rich 1H-Tetrazole (CH_2_N_4_) under High Pressure

Wenbo Li, Xiaoli Huang, Kuo Bao, Zhonglong Zhao, Yanping Huang, Lu Wang, Gang Wu, Bo Zhou, Defang Duan, Fangfei Li, Qiang Zhou, Bingbing Liu, and Tian Cui*

State Key Laboratory of Superhard Materials, College of physics, Jilin University, Changchun, 130012, P. R. China

Correspondence and requests for materials should be addressed to T.C. ([cuitian@jlu.edu.cn](mailto:cuitian@jlu.edu.cn))


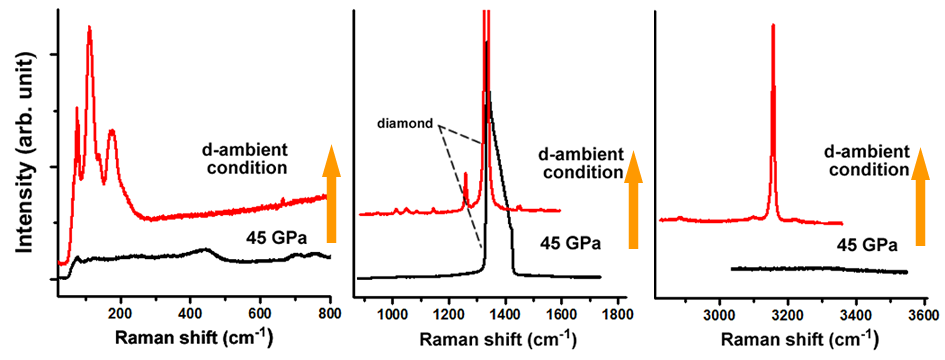


**Figure S1**. Raman spectra are reversible when sample (1H-tetrazole) decompresses from the maximum pressure 45 GPa to ambient conditions.
